# Diagnosis-Based Hybridization of Multimedical Tests and Sociodemographic Characteristics of Autism Spectrum Disorder Using Artificial Intelligence and Machine Learning Techniques: A Systematic Review

**DOI:** 10.1155/2022/3551528

**Published:** 2022-07-01

**Authors:** M. E. Alqaysi, A. S. Albahri, Rula A. Hamid

**Affiliations:** ^1^Informatics Institute for Postgraduate Studies (IIPS), Iraqi Commission for Computers and Informatics (ICCI), Baghdad, Iraq; ^2^Department of Medical Instruments Engineering Techniques, Al-Farahidi University, Baghdad 10021, Iraq; ^3^College of Business Informatics, University of Information Technology and Communications (UOITC), Baghdad, Iraq

## Abstract

Autism spectrum disorder (ASD) is a complex neurobehavioral condition that begins in childhood and continues throughout life, affecting communication and verbal and behavioral skills. It is challenging to discover autism in the early stages of life, which prompted researchers to intensify efforts to reach the best solutions to treat this challenge by introducing artificial intelligence (AI) techniques and machine learning (ML) algorithms, which played an essential role in greatly assisting the medical and healthcare staff and trying to obtain the highest predictive results for autism spectrum disorder. This study is aimed at systematically reviewing the literature related to the criteria, including multimedical tests and sociodemographic characteristics in AI techniques and ML contributions. Accordingly, this study checked the Web of Science (WoS), Science Direct (SD), IEEE Xplore digital library, and Scopus databases. A set of 944 articles from 2017 to 2021 is collected to reveal a clear picture and better understand all the academic literature through a definitive collection of 40 articles based on our inclusion and exclusion criteria. The selected articles were divided based on similarity, objective, and aim evidence across studies. They are divided into two main categories: the first category is “diagnosis of ASD based on questionnaires and sociodemographic features” (*n* = 39). This category contains a subsection that consists of three categories: (a) early diagnosis of ASD towards analysis, (b) diagnosis of ASD towards prediction, and (c) diagnosis of ASD based on resampling techniques. The second category consists of “diagnosis ASD based on medical and family characteristic features” (*n* = 1). This multidisciplinary systematic review revealed the taxonomy, motivations, recommendations, and challenges of diagnosis ASD research in utilizing AI techniques and ML algorithms that need synergistic attention. Thus, this systematic review performs a comprehensive science mapping analysis and identifies the open issues that help accomplish the recommended solution of diagnosis ASD research. Finally, this study critically reviews the literature and attempts to address the diagnosis ASD research gaps in knowledge and highlights the available ASD datasets, AI techniques and ML algorithms, and the feature selection methods that have been collected from the final set of articles.

## 1. Introduction

Autism spectrum disorder (ASD) is a neurodevelopmental disorder that affects a person's interaction, communication, and learning skills. Autism was first characterized by Leo Kanner in 1943, who tried to understand the relationship between autism and sociodemographic factors such as social class, sex, maternal education, age, and race [1–3]. According to the World Health Organization (WHO), every year, out of 160 children worldwide, one child is diagnosed with autism [4]. In developed countries, about 1.5% of children were diagnosed with ASD in 2017 [5]. Recently, autism research has gained more attention, including the early diagnosis process and investigation of affected features of this disease. Diagnosing is complicated and is a complex and arduous process due to the significant differences in the types and severity of symptoms [6–12]. Sometimes these responses are difficult to distinguish between very young children without autism and with autism which further complicates the diagnosis task. Symptoms of autism generally appear in the first two years of life and develop over time [13]. However, doctors and medical staff consider diagnosing children with autism a complex task in the first two years. Although many clinical tools for detecting ASD early, they are cumbersome diagnostics and are not often used unless there is a significant risk of developing ASD [14]. A diagnosis of autism can come at any age, so early detection can make a big difference in treating it later [15]. Therefore, early detection can improve a child's skills through treatment [4]. This has encouraged researchers to investigate new solutions for the early diagnosis of autism, as they help recover and improve treatment [16]. Accordingly, the introduction covers three important questions according to our study's scope and provides an appropriate answer. The first question that should be discussed is “What is the main cause of ASD?”

ASD has no single known cause. Given the complexity of the disorder and the fact that symptoms and severity vary, there are probably many causes. Both genetics and environmental factors may play important roles. Many different genes are involved in ASD for genetic reasons, such as Rett syndrome or fragile X syndrome [17]. In addition, genetic changes (mutations) may increase the risk of autism as some genetic mutations appear to be inherited. In contrast, others occur spontaneously [17].

On the other hand, environmental factors such as viral infections, medications, or complications during pregnancy play a role in triggering ASD. Also, they may be caused by familial or sociodemographic characteristics, such as race, gender, and age, or they may be behavioral factors implying a person's behavior (introverted, not dealing with peers, etc.) [18]. In addition, there is no way to prevent ASD, but early diagnosis and intervention are most helpful and can improve behavior, skills, and language development. However, intervention is beneficial at any age. There is no consensus on the reasons underlying the phenomenon of more boys than girls being diagnosed with ASD, albeit sexual differences in the brain exist. Boys and girls are also known to have different levels of androgens and testosterone and estrogen hormones. However, no clear explanation accounts for the sexual differences in susceptibility [19]. In these contexts, there is no complete knowledge of the causes of this disease, which motivates researchers to diagnose patients in many directions. With regard to these directions, with the advancement of artificial intelligence (AI) and machine learning (ML), autism traits can be improved diagnostic predictions at a pretty early stage using different techniques. Therefore, many researchers have noted that AI and ML play a significant role in the early diagnosis of autism because it helps clinicians shorten the diagnosis process and obtain more accurate results [20–24]. This motivated us to ask the second question, “What are the current directions of autism diagnosis based on AI and ML?”

Scientific studies, equipment, and test tools contributing to the early ASD diagnosis will also provide excellent support for controlling and treating this disease. At present and after the significant development in computer science and information technology, ML has been used to diagnose and analyze various conditions such as heart disease, lung cancer, diabetes, and COVID-19, which effectively classify and predict various medical fields. In these contexts, diagnosis of ASD used many methods for ML such as k-nearest neighbors (KNN), naive Bayes (NB), Random Forest (RF), and Support Vector Machine (SVM) and AI techniques (deep learning) such as convolutional neural network (CNN) and Recurrent neural network (RNN). Evaluate algorithms by metrics vary from one researcher to another.

Several trends have emerged in identifying, diagnosing, and analyzing autism using AI and ML. First, magnetic resonance imaging (MRI) is a cross-sectional image of the brain and a medical imaging method to clarify pathological changes in living tissues [25, 26]. MRI limitations can be reported as expensive and unavailable in many hospitals, especially in resource-poor countries. Besides, the MRI scanner is a small, enclosed space; it can cause claustrophobia and sometimes difficulty fitting, increasing the complexity of diagnosis, especially for this acute disease. Also, a magnetic field can affect metal devices if implanted in the body. Second, an electroencephalogram (EEG) is a test that detects the electrical activity in the brain by using tiny metal discs (electrodes) attached to the scalp. The main disadvantage of EEG recording is poor spatial resolution. In addition, it does not offer the highest accuracy for the diagnosis of ASD. Third, sociodemographic diagnosis is based on sociodemographic features (such as sex, age, and race), and diagnosis of this type alone is considered inadequate. So, it does not give high accuracy, and medical examinations (blood tests, hearing tests, and genetic tests), in general, cannot be ignored.

In conclusion, each diagnostic direction has limitations regarding the utilized diagnosis features. Each approach alone does not provide an accurate diagnosis procedure. These directions could not have been realized the proper diagnosis process using specific features and neglecting others. Yet no new path brings them together in an integrated diagnosis by AI and ML methods. Therefore, the question arises about “What is the expected research direction to improve the diagnosis of ASD?”

When the integration between these paths is introduced, it strengthens and supports the diagnostic process. According to the literature, there is no integration between these paths of medical features and sociodemographic features in the context of AI techniques, ML, and deep learning algorithms, which motivates this study. This aspect reinforces the importance of medical characteristics, especially their integration with sociodemographic factors. Although, in the literature, there is no integration of medical features with sociodemographic features. Medical aspects give accurate results such as examining vitamins (D3, ZINC, B12, etc.) that cause affect autistic children. As was not covered based on medical and laboratory due to the lack of laboratory or medical tests (such as a blood test or others), ASD diagnosis may be challenging. Thus, an extensive investigation of the diagnosis of ASD towards hybrid multimedical test features and sociodemographic characteristics using AI and ML techniques as a systematic review needs to be presented.

## 2. Methods

**Figure 1:**
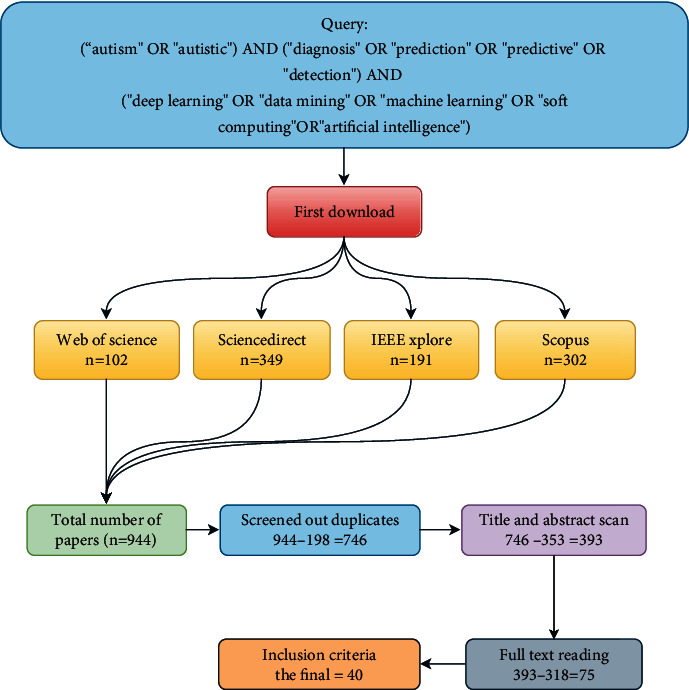
Study selection flowchart including the search queries and inclusion criteria.

**Figure 2:**
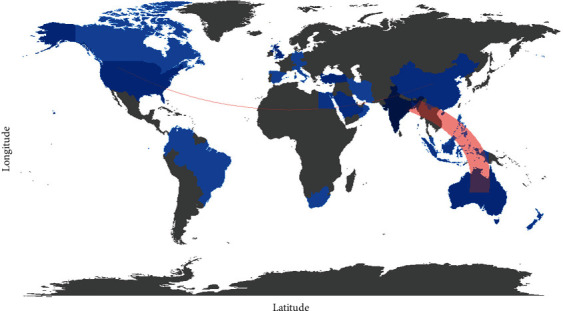
Collaboration world map.

This study used a style of systematic reviews and meta-analyses guidelines illustrated in Figure 1 [27–32]. In literature reviews, researchers must search more than one database, not just one related database, to cover the most related articles. In this study, four databases were selected, which are considered the most reliable: (1) Web of Science (WoS), which allows access to different articles in different domains; (2) Science Direct (SD), which provides access to a range of journals from various scientific domains, including medicine, science, and technology; (3) IEEE Xplore digital library, which allow various engineering and technology-related publications; and (4) Scopus, which offers massive coverage of literature from all disciplines [33–38]. Based on their academic reliability and presentations from different academic disciplines, these databases were selected. A comprehensive search was conducted for English-language papers from 2017 to 2021. These indices were selected for their enough coverage. Most of the studies are related to our research, considering the trend toward a different diagnosis in the medical and sociodemographic features of ASD using different AI techniques, data mining, ML, and deep learning algorithms. We searched using a query consisting of disease-specific keywords, as shown in Figure 2.

### 2.1. Inclusion Criteria

1. Studies in English journals or conference papers
2. Studies related to various contexts such as diagnosis, detection, and prediction for autism use different methods, algorithms, and AI techniques to optimize ASD processes and enhance diagnosis decision-making
3. The above contexts were conducted based on medical laboratory testing, social demography, family characteristic features, and criteria based on questionnaires

### 2.2. Exclusion Criteria

1. Articles that monitor ASD based on play therapy
2. Articles that monitor ASD using smart sensors and robotics
3. Articles that diagnosis based on medical image processing using MRI
4. Articles that diagnosis based on EEG signal processing

### 2.3. Data Extraction and Classification

After duplicate papers are removed, the next step includes scanning papers' titles and abstracts according to the inclusion/exclusion criteria. A full-text reading is achieved for potentially related articles. The surveyed works and a running classification for all the articles allow us to create the proposed taxonomy. In the current study, various data were collected and extracted in articles related to autism, which in turn led to a deep perception in the researchers' narrative of things related to the purpose of the article and the motivation and challenges faced by the researcher, also the limitations or recommendations that we will address later in the subsequent sections, in addition, the number of features and algorithms used and the method in which the researcher decided to diagnose or identify autism and assessment matrices, which in turn serve to determine the efficiency and quality of the algorithm used.

## 3. Comprehensive Science Mapping Analysis

Five comprehensive analysis processes have been presented based on medical/laboratory features and sociodemographic features in this section for the articles collected from the four databases based on the objectivity of the papers based on:

### 3.1. Collaboration World Map

This section discusses the articles' distribution on ASD in single or multiple publications in each country, in this regard, as can also aim to observe networking and collaboration between countries. Besides, in Figure 2, the highest studies in Bangladesh and India have thirteen articles, followed by the United States, which have eight articles. In addition, Australia and Germany have each obtained six articles. Furthermore, Turkey, China, and the United Kingdom obtained four articles. Likewise, Saudi Arabia, Indonesia, New Zealand, and Spain have obtained three articles. The countries that submitted two papers were Canada, Ecuador, Egypt, Italy, Iran, Philippines, and South Africa. While the countries that contributed little as one research are Austria, Brazil, Colombia, Malaysia, Oman, and Venezuela, which can depict the global collaborations. The blue color on the map represents research cooperation among nations. Additionally, the pink border linking the countries indicates the extent of cooperation between the authors. It is interesting to see how countries with the most publications on detecting and treating ASD have engaged in such partnerships. India and Australia on one side and the United States of America and China on the other side have engaged in the most significant collaborations with countries that are sometimes very distant from each other and therefore have the collaboration can lead to sharing of policies to reach the development in scientific ASD research.

### 3.2. Word Cloud

**Figure 3:**
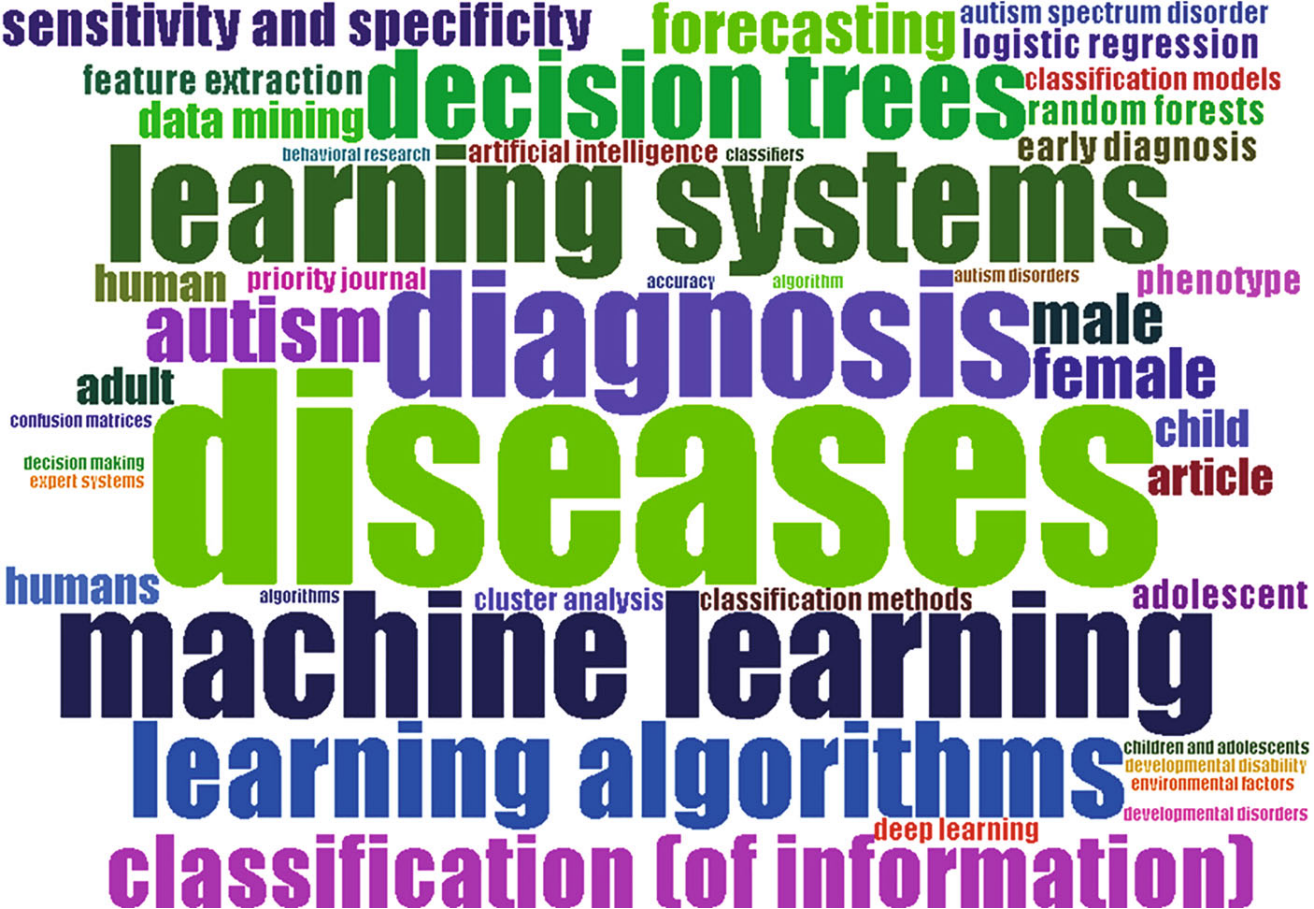
Word cloud.

A word cloud describes the focus of detail of these studies and the types of data used in the articles. In addition, it highlights the essential subjects associated with a specific topic. Word clouds are a way of visually representing a set of words. In addition, word cloud summarized many keywords of studies in the form of an easy and understandable ASD picture. It creates an image of a given set of frequently used words. Most studies focused on ASD patients' characteristics (autism, diagnosis, diseases, machine learning, learning algorithm and autism spectrum disorders, etc.). Words are highlighted by making them more central in the picture or increasing their font size.  Figure 3 shows a word cloud based on the 44 most common keywords used.

### 3.3. Historical Direction Citation Network

**Figure 4:**
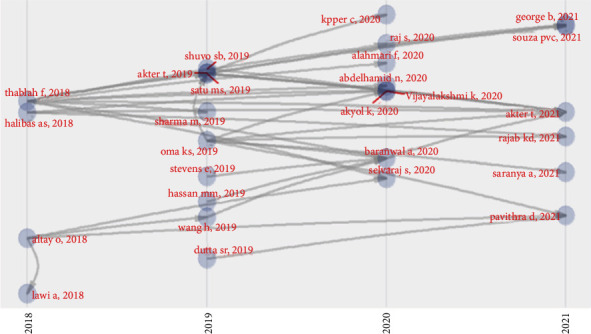
Historical direction citation network.

The proposal represents a chronological network map, a historiography map for the most relevant direct citations resulting from article collection. The citation network technique provides the scholar with a new work method that may significantly affect future historiography, as shown in Figure 4.

### 3.4. Word Growth

**Figure 5:**
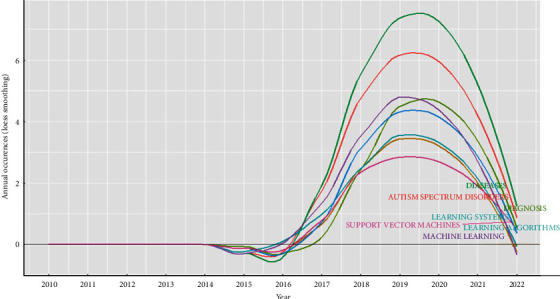
Describe word growth.

Figure 5 Indicates word dynamic analyses. The cumulated occurrences of the generated keywords plus present the shift in the research terms in the last five years. The most frequent terms are diseases, ASD, diagnosis, learning system, Support Vector Machine, learning algorithm, machine learning, and decision tree. Figure 5 illustrates the relation for all most keywords utilized with the year of the article, whereas the peak of the figure was (diseases) in 2019 and the bottom was (SVM).

### 3.5. Conceptual Structure Map

**Figure 6:**
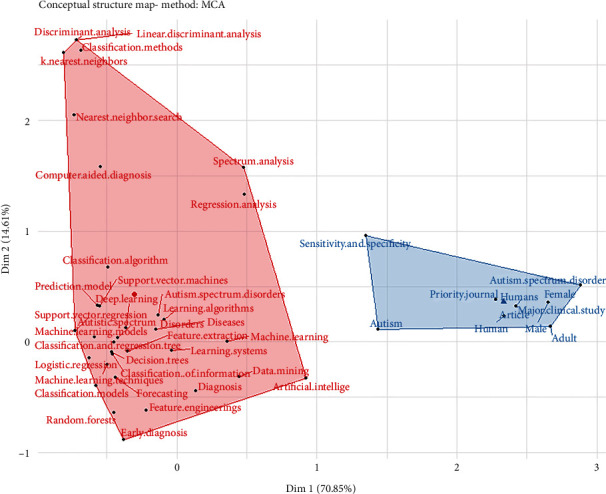
Conceptual structure map.

The factorial analysis of the survey can be presented in Figure 6. There are two groups of words used in this survey among researchers.

## 4. Taxonomy Results

**Figure 7:**
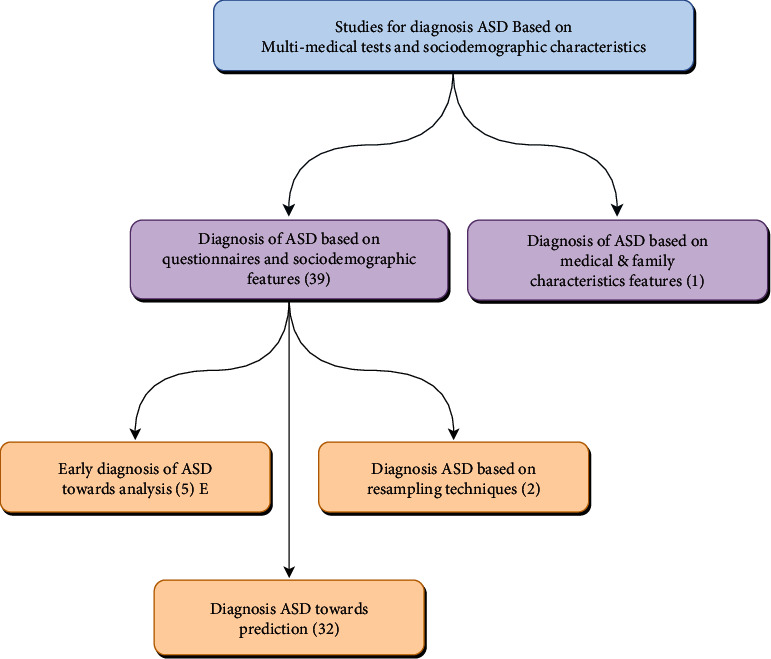
Taxonomy of research literature on diagnosis of ASD.

The purpose of using taxonomy in the ASD field is to understand the philosophy of the final set of articles and determine their analytical dimension to link them theoretically and intellectually on a systematic basis, which makes it easier for the medical staff to visualize to understand the ASD research in a smooth way away from the complexity and overlap in the understanding of AI theories in general. Accordingly, the collecting articles begin with 944 papers from the four databases. Upon the completion of duplication screening, a total of *n* = 198 papers were removed, resulting in a total of *n* = 746 articles. Then, the screening was meant for the title and abstract scanning, which resulted in a total of *n* = 393 articles. The following filtration was conducted through a full-text reading for the identified articles and resulted in a total of *n* = 75. The articles (*n* = 40) were screened and determined as relevant to the review based on our inclusion and exclusion criteria. The selected articles were divided based on similarity, objective, and aim evidence across studies. They are divided into two main categories: the first category is “diagnosis of ASD based on questionnaires and sociodemographic features” (*n* = 39). This category contains a subsection that consists of three categories: (a) early diagnosis of ASD towards analysis, (b) diagnosis of ASD towards prediction, and (c) diagnosis of ASD based on resampling techniques which we will address later. The second category consists of “diagnosis ASD based on medical and family characteristic features” (*n* = 1). The papers were divided and designed based on overlap and content in a solid taxonomy, as shown in Figure 7.

### 4.1. Diagnosis of ASD Based on Questionnaires and Sociodemographic Features

This category contains (39/40) articles and includes three sections. A questionnaire assesses the diagnosis, and treatment is one of the standard diagnostic methods a doctor and medical staff carry out in diagnosing children with autism based on a set of questions. The collection of questions given to parents to answer includes inquiries related to race, age, family history, etc., based on family and social characteristics (social demographic) that play a role in diagnosing and exploring the disease. In addition, Autism Diagnostic Observation Schedule (ADOS) and Autism Diagnostic Interview-Revised (ADI-R) are questionnaires related to the behavior and action of the child and asked the parents to be analyzed and studied [39, 40]. Although there are many social characteristics in this aspect, each factor has its weight that represents the importance and the assessment according to the physician's viewpoint.

#### 4.1.1. Early Diagnosis of ASD towards Analysis

This section consists of (5/39) articles. Early detection of ASD disease (i.e., mental and neurological disorders) is the primary step in treating the autism patient to be recovered. The early diagnosis of ASD through AI techniques focuses on detecting or predicting autism patients as early as possible to have the best chance for successful treatment. In addition, recent research has proven that early detection of autism leads to the furry assistance provided by doctors, making it easier for the patient to recover faster. In [4], the study is aimed at estimating ASD at a sooner possible time and increasing accuracy, reducing medical costs. The final goal is to create an online tool that can provide ML-based analysis to a user to detect autism early. In [14, 15], the utilized early detection ASD datasets of different stages of life (toddler, child, adolescent, and adult) have been conducted and the analyzed results of using a range of varying ML classifiers to explore the significant features of ASD. A lot of children in the world become autistic for some undefined factors. The main objective of the research [1] is to analyze individual features which cause autism by using the AI technique. If individual factors are identified and checked for their severity, it is possible to prevent autism across the globe. A diagnosis process has been achieved in the study of [5] by integrating data from many diverse sources, such as medical or intervention centers, hospitals, and academic centers. It can facilitate the early diagnosis of autism patients.

#### 4.1.2. Diagnosis of ASD toward Prediction

This section consists of (32/39) articles. Recently, ML has been extensively applied in many fields of life and medical domains [40–42]. ML can predict and detect ASD, which in turn helps the medical staff in diagnosing autism and facilitates the work of the doctor [13, 39, 43, 44]. Therefore, ML classifiers can predict the presence of autism attributes in a person of any age, which helps in the faster recovery process [45]. In [46], the Logistic Regression Model includes data and feature engineering, model training, and model testing has been presented. The quality of the prediction depends on several criteria determined by the expert in addition to algorithms in which evaluation metrics define accuracy as the most powerful performance. The purpose of research in [19] is to identify factors that were unknown features for autism diagnosis but that have been reported and investigated by clinical studies in the literature as contributing and/or comorbid factors of ASD. The main contribution of studies [6, 47, 48] is that the exploration of ASD using classification algorithms trained with a set of attributes produces outstanding prediction results based on sociodemographic features. The studies [18, 49–52] were aimed at utilizing feature selection to determine common influential attributes that are usually selected by feature selection methods and have a direct impact on the classification performance of predicting/screening tools, which shows a comparison of the performance of different prediction algorithms to diagnose ASD, which uses more than one algorithm and determines the best model based on the accuracy [53–56]. In [57], data analysis is presented to determine the relationship between the attributes and the degree of correlation for each feature with others based on sociodemographic features. Despite the ability of traditional algorithms (ML) to predict well, the utilization of neural networks has achieved practical results in prediction and diagnosis [58].

#### 4.1.3. Diagnosis of ASD Based on Resampling Techniques

This section consists of (2/39) articles. The concept of resampling techniques and data imbalance have been covered in this section. One of the methods commands utilized in ML is the balancing of data based on the target class, which in turn achieves high accuracy in the classification process and gives a perception closer to reality [53]. It has been noted in some articles that specialize in screening ASD that there is an imbalance or almost inequality in the data presented, which leads to a decrease in the accuracy of the diagnosis disease. So, when the data is processed by an ML, such as classification techniques, models derived tend to favor the most frequent class labels, and the low frequent class labels get undermined [16]. Consequently, models are biased, and accuracy can no longer be used to measure integrity. Methods of resampling techniques that lead to a normal distribution of ASD data improve accuracy in the prediction of autism and avoid the problem of data heterogeneity.

#### 4.1.4. Diagnosis of ASD Based on Medical and Family Characteristic Features

This category contains 1/40 obtained by searching the four trusted databases regarding the medical and familial characteristics of individuals with autism. This is considered a gap that should be addressed and extensively covered. The authors of [59] dealt with the diagnosis and prediction of autism using ML algorithms based on medical and family characteristics. Therefore, facilitate access to ASD knowledge and support professionals and physicians in their clinical decisions by an ontology-driven decision support for autism diagnosis and treatment. As can also focus on the diagnostic process, the patterns and constructed rules from the ML model enable physicians and experts to diagnose.

## 5. Discussion

This section targets to highlight and debate three basic concepts after extracting information from the collected articles: (1) the motives, benefits, and the importance of the topics that made researchers highlight and try to provide solutions to solve problems; (2) the challenges and what current and former researchers face in the cases and obstacles that have been reported; and (3) the recommendations recommended by the authors and what is the future work to be applied later in the ASD research path.

### 5.1. Motivations

**Figure 8:**
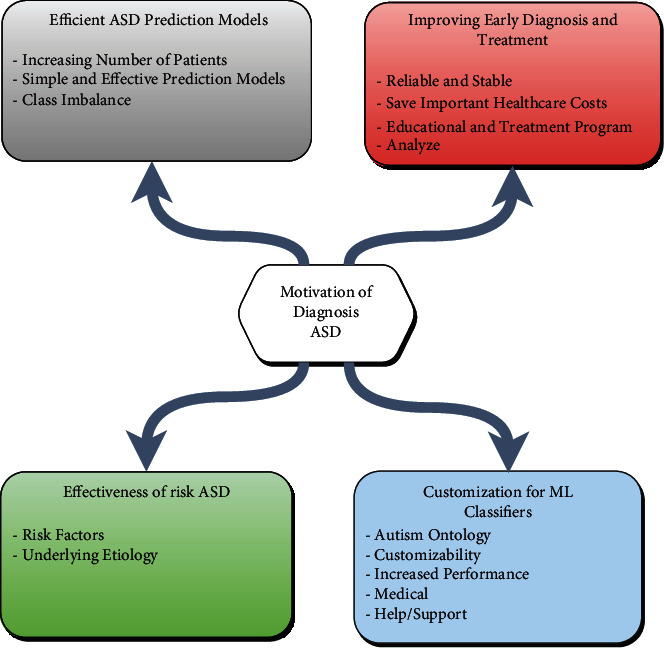
Motivation categories of diagnosis ASD.

The significant increase in ASD prompted researchers to find ways to diagnose, predict, and improve treatment for this disease. Motives fall into four categories, as indicated in Figure 8.

#### 5.1.1. Improving Early Diagnosis and Treatment

The diagnosis of ASD is essential, and without a diagnosis, this can make so many areas of life complex, distressing and bewildering for the undiagnosed person. This sort of autism spectrum syndrome is detected a great deal later than is conceivable [60]. Detecting ASD at an early step also prevents the affected condition from further deteriorating [45]. ASD must be diagnosed early to be more reliable and stable [4, 61]. On the other hand, early diagnosis has a significant impact on intervention and treatment [47, 50, 53], decreasing and saving necessary healthcare costs [46, 62]. Furthermore, it provides mental improvement for overall health [52]. In [40], providing an appropriate educational and treatment program is one way to reduce the condition that the patient suffers from [42]. As a result, AI algorithms have been widely applied in many fields to achieve better results for prediction and diagnosis [58, 63]. Although there is virtually no cure for this disorder [14], this factor has motivated researchers to analyze the disease and explore to improve its treatment methodology [16, 41].

#### 5.1.2. Customization for ML Classifiers

A classifier is an ML algorithm used to assign a class label to data input. A classifier utilizes some training data to understand how given input variables relate to the class. Regardless of increased performance in diagnosing ASD applied to different ML algorithms of available clinical tests [64], this led to generating a motivation to develop algorithms and reach the highest results in terms of accuracy and use even hybrid algorithms. In these contexts, many known data mining algorithms have been used or developed to solve this issue [56], such as the Bayesian fuzzy neural network proposed that was used in the study [65]. In addition, the customization of the ML allowed the merging of data from many varied sources, such as medical or intervention centers, hospitals, and academic centers, with help/support [5]. The authors' primary motivation in [59] recommended autism ontology, which is the major urge for developing algorithms based on subfield and is mainly used for calculating precision, pace, and customizability. Increased performance for the ML classifiers in diagnosing ASD was addressed using different algorithms regardless of available clinical tests [57]. This led to generating a motivation to develop algorithms and reach the highest accuracy results and even use hybrid algorithms using data mining algorithms [56].

#### 5.1.3. Effectiveness of Risk ASD

Various risk factors play a significant role in individuals with ASD, such as family and patient medical history. In addition, as can be to check gender differences, the disparity in ASD diagnosis between males and females suggests differences in a big underlying etiology of autism [19]. One step that could be taken toward using artificial intelligence and machine learning is to identify the different types of ASD risk factors [19]. In addition, the role of genetic and environmental factors is of great importance in its pathophysiology [52].

#### 5.1.4. Efficient ASD Prediction Models

Due to the increasing number of patients, the authors recommend that there are serious demands to implement simple, effective, and more accurate prediction models [15, 51]. Furthermore, there is a requirement to develop fast medical diagnostic systems using the integration of ML methods [44]. AI algorithms have been used to save costs for human diagnosis and improve the quality of prediction [54, 55, 66]. ASD prediction can facilitate the time and effort of the medical staff based on the efficiency of the prediction model. On the other side, there are some articles about the impact of class imbalance on classification models using a real dataset related to ASD screening which led to generating an incentive for researchers in this field [67, 68].

### 5.2. Challenges

**Figure 9:**
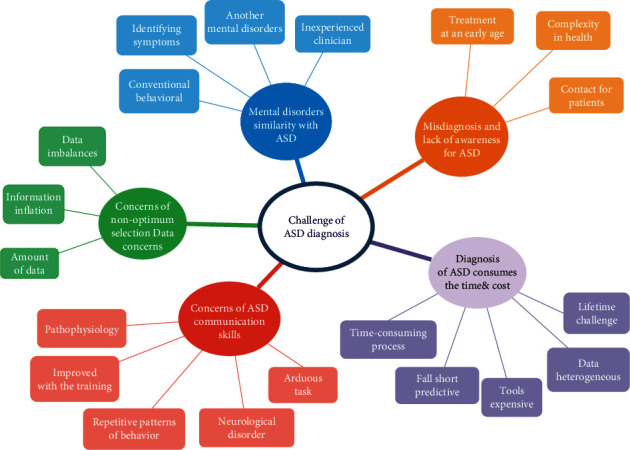
Challenges of ASD diagnosis.

In this study, the challenges that researchers faced in classifying or predicting ASD have been addressed. They were divided into five clusters, and each cluster contained similar articles as can be seen in Figure 9.

#### 5.2.1. Mental Disorders Similar to ASD

Many mental disorders that are similar to ASD become a challenge as there are many other mental disorders whose few displays are very similar to those with ASD symptoms, making prediction a challenging task [16]. In addition, the difficulty of identifying these symptoms for adults is more complex than for children. ASD is complicated to uncover and diagnose by conventional behavioral studies [14]. Occasionally, owing to the overlapping nature and similarity of symptoms, it might be difficult for an unskilled practitioner to make an accurate diagnosis. Numerous instances of incorrect and late diagnosis of illnesses have cost individuals money, time, and sometimes patients' lives [66].

#### 5.2.2. Misdiagnosis and Lack of Awareness for ASD

People with ASD experience various types of symptoms, for example, difficulty interacting with others, repetitive behaviors, and difficulty functioning correctly in other areas of day-to-day life. These symptoms generally occur in early childhood. Most people are not well aware of the syndrome; therefore, they do not know whether a person is suffering from the disorder or not [47]. Many parents cannot express all the symptoms or behaviors their child is experiencing [6]. General practitioners (GPs) and family physicians are typically the first stages of contact for patients or family members concerned with ASD features noted in themselves or their family members [49]. Unfortunately, some families and adult patients are insensible of ASD features that may be exhibited [49]. That is why these children are not able to have proper treatment at an early age, which causes more complexity in their health [4]. Furthermore, this highly complex research environment makes it even harder for practicing physicians and primary care providers to keep up with recent advances [59].

#### 5.2.3. Diagnosis of ASD Consumes the Time and Cost

Autism poses an emotional lifetime struggle for numerous families because of the nature of the disorder [50]. In comparison to ordinary persons, ASD patients have problems during early development. Multiple instruments and clinical and nonclinical procedures have been applied. However, it takes an extended period to obtain a thorough diagnosis [55]. This implies that diagnostic techniques should be altered. Conversely, the data used for ASD screening is heterogeneous and multisource, resulting in existing screening tools for ASD screening being expensive, time-intensive, and sometimes falling short in predictive accuracy [48, 51, 62]. The observations of (ASD) depend on the behavioral evaluation of the patients, which takes more time and effort [44], especially since it is a complicated, time-consuming process that is particularly challenging in older individuals [39] to detect traits through screening tests [67].

#### 5.2.4. Concerns of ASD Communication Skills

ASD causes deficits in cognition, communication, and social skills. ASD is a neurodevelopmental disorder characterized by deficits in social communication and social interaction and the presence of restricted, repetitive behaviors [1, 61]. Three distinct behavioral conduct deficits define ASD: (1) social interaction deficits, (2) verbal and nonverbal communication skills impairments, and (3) the prevalence of recurrent nonfunctional activities. At the moment, no one brain or psychological explanation is capable of accounting for all three deficiencies concurrently [19, 46, 64]. ASD is a heterogeneous disorder [41]. Thus, it faces abnormal communication challenges [15]. Children with ASD require extra care [58]. Furthermore, ASD is a chronic condition in which the individual's quality of life can be enhanced via training [40, 43]. People diagnosed with ASD have a range of symptoms, and that is why it is termed a “spectrum” disorder. Since ASD is a neurological developmental disorder, there is no specific medical test, whereas its symptoms may vary from one person to another [42, 54]. The reason for ASD is still under research for healthcare [18, 45]. Thus, making the diagnosis of ASD an arduous task [57]. The study of [5] focused on screening issues for autism based on behavioral features.

#### 5.2.5. Concerns of Nonoptimum Selection Data Concerns

The quick and continuous increase in information inflation and information technology in world databases is doubling every year. This caused making processing operations on this amount of data a challenge [56]. The trouble related to medical applications like (ASD) data imbalances in which situations are more than just controls in the dataset [68] may affect the accuracy of detection [60]. ML techniques face problems as they directly impact the classification model's performance concerning selecting the best dataset. In an imbalanced dataset, most of the instances belong to one class; this leads to class imbalance being a complex problem that, if untreated, may lead to biased results [67].

### 5.3. Recommendation and Future Work

This section is aimed at discussing recommendations linked to the diagnosis of ASD. The guidance and future works recommended by the authors are listed in bullet points:

1. In the study [59], the authors recommended giving ideal decisions to doctors through good diagnosis and treatment, which in turn finds the best treatment method in addition to creating a road map by finding strengths, weaknesses, and services required based on factors, patient symptoms, and family medical history
2. The main recommendation [19], extensive research on the etiology of ASD, and potential avenues for prevention and treatment can make lives easier for families and caregivers
3. Five particular pathways in treating ASD patients have been presented in [41]. The authors of this study recommended that an extensive comparison be used for training the ML model with a more extensive and more appropriate set of data
4. Collecting as many possible data from different sources can boost the accuracy performance [4, 5, 13]. Finding similar significant features from individual datasets [15] can analyze more data to improve the detection of ASD [14]
5. In the article [1], utilizing more historical, survey, and web data can provide more justification for the obtained results with different validation techniques
6. As can collect more samples to decrease the impact of special samples on neural network training, the introduction of many factors affecting autism on the networks increases the accuracy of diagnosis and prediction. Use some ways to discover and eliminate noise samples [47]
7. Because of the efficiency of the Multivariate Curve Resolution (MCR) model, a better autism detecting model can be given to society as a potentially very effective automated international recommender system. This can detect autism at an earlier stage for the proper medical guidance and fast recovery. Besides, it can be extended for behavioral and sociodemographic data analysis on other datasets with implementation [44]
8. Deep learning algorithms can be utilized, and helping medical staff makes more accurate decisions on autism [45]
9. The ML classification can be affected by data imbalance on unstructured and more complex acoustic data features of autism [67]
10. Investigate differences in more specific clinical comparison groups (e.g., personality disorders, anxiety disorders, other developmental disorders) and specific gender groups [6]. In addition, studies on ASD in certain countries or at specific ages may also be conducted [65]
11. Improvement of multilayer fuzzy cognitive map MFCM-ASD through introducing new layers to evaluate ASD can represent new dimensions of symptoms to be included in the diagnostic process [40]

## 6. Diagnosis ASD: A Critical Analysis of the Literature

Three complex and important section analyses associated with the current topic of this study are stated in this section.

### 6.1. Available ASD Datasets Used with ML and AI

In this section, the insertion data from the articles were included in five important columns and presented in Table 1. The extraction of the information consists of (1) the source of the data obtained; (2) the number of the dataset used, which may be one or more than one used by the author; (3) the size of the data that represents patients whenever the larger the number of data and the higher the accuracy of the classifier; (4) the number of attributes/features used in each dataset and type of attributes varies from one data to another, in addition to the author's view of the use of specific attributes, which is supposed to give the researcher justification in using these attributes and whether they are sufficient or not that represent as (a) questionnaires are features obtained from parents, (b) sociodemographic features that represent race, age, gender, etc., and (c) medical features; and (5) the associated task which specifies the method used (classification or clustering).

All of these data mentioned in Table 1 play an essential role in the accuracy of ASD classification. Each element can affect directly proportional to the other in the ML model. However, suppose a comparison is made for the weight of each study. In that case, the large bulk of the number of features/attributes and the size of the dataset will be best because most of the limitations that most researchers faced, according to the literature, were the smallness of the data and the number of attributes choosing the suitable features for classification.

### 6.2. Utilization of ML Methods and AI Techniques

AI science and ML algorithms are implemented with multidisciplinary studies that aid in building more precise results. Thus, several articles on autism utilized ML methods have been presented in Table 2.

It can be seen in Table 2 the information related to datasets extracted and established in the literature, such as algorithm techniques used and how evaluation by metrics (accuracy, specificity, sensitivity/recall, F1 measure, AUC, precision, TPR, and FPR). This data can provide valuable information, especially in developing new methodologies and selecting one of the case studies in this table. Through literature reviews, the algorithms used in this literature were obtained and listed in Table 2, and these algorithms were evaluated according to the metrics used in each article. Most of the reviewed studies expected high accuracy and performance, although the results obtained from these techniques varied from one study to another. In addition, there are various evaluation metrics regarding the type of the studies. The accuracy has been widely used, while other metrics such as precision, TPR, and FPR have obtained little attention without justifying that. The comprehensiveness and integration of the most used evaluation metrics (which means using a wide range of metrics) for the prediction of ASD is what gives precise results and objectivity to autism work.

**Figure 10:**
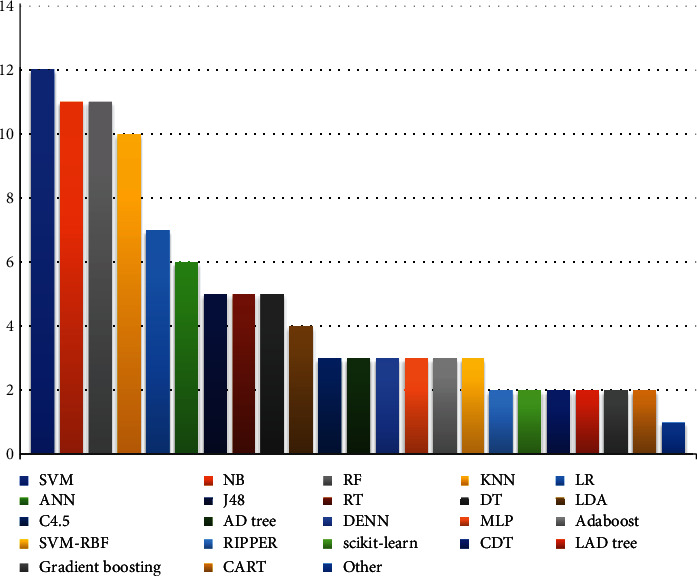
Description of ML algorithms used in the literature.

Furthermore, the below Figure 10 shows the number of used algorithms according to the current ASD research, while the SVM algorithm was utilized 12 times more than the rest of the algorithms, followed by NB and RF algorithms, which were used 11 times. The efficiency of the SVM algorithm is obtaining the highest results. We also point out other (C5.0, ID3, DL, Decision table, K-star, RNN, LMT, NP Tree, DS, Firefly Algorithm-Random Forest, RF (hyperparameter), DT with LR, XGBOOST, SGD, ELM, REP Tree, FDA, RF (CART), DT (CART), GLMBOOST, RF(CART)+RF(ID3), KBS, SL, Apriori, CARMRMR, SVM-NP, PART, SVM-PK, SVM-PUK, SMV-Liner Kernel, MCR, QDA, APR, Worm optimized extreme learning machine (WOEM), Bayesian fuzzy neural network (BFNNRELU), and improved adaptive neuro-fuzzy interference system (IANFIS) that it is a set of algorithms that have been utilized only once.

### 6.3. Utilization of Feature Selection Methods

**Figure 11:**
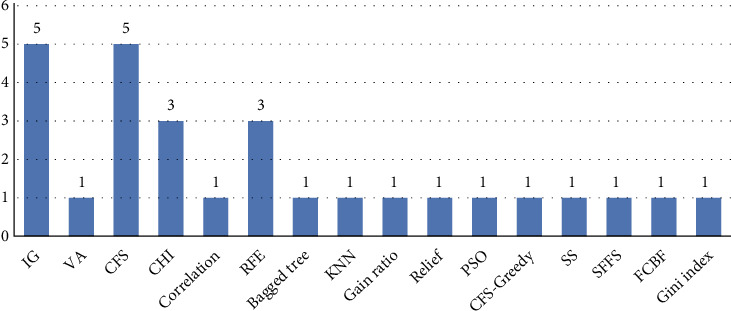
Description of features selection methods used in the literature.

Selecting features remains an essential task before developing a predictive model for ASD classification.  Figure 11 indicates the number of iterations utilized in feature selection for the 40 articles. The concept of feature selection consists of three filtering methods, wrapper and embedded.

As shown in Figure 11, the filtering method was frequently used more than others. Besides, the info gain (IG) and correlation feature selection (CFS) are more utilized than the rest of the feature selection. The researchers indicate the quality of this feature when using it and obtaining effective results. The main benefit of choosing a feature selection is identifying and choosing the essential features that represent the bulk or great weight in choosing the right features, which reflects the accuracy of the results.

## 7. Conclusion

This work is aimed at comprehensively reviewing the literature on autism, which can help the most significant number of researchers and people in this field, especially discovering that ASD diagnosis is a high cost and consumes time and effort. This study provided detailed descriptive information concerning the detection and prediction of ASD that interact with the disease classification covered in the literature in the context of AI techniques and ML algorithms contributions. We comprehensively analyzed some studies to highlight the benefits, challenges, and recommendations related to ASD and found specific gaps. In this systematic review, the focus has been dedicated to the AI techniques and ML and how they can integrate autism diagnosis into the concept of using multimedical tests and sociodemographic characteristics. Several feature selection approaches and ML algorithms have been used in the literature without suggesting the hybridization among both. The feature selection approaches of multimedical test features and sociodemographic characteristics based on reliable guidelines can be an essential method to be applied in ASD classification models to obtain a quick and specific diagnosis. Classification models based on feature selection approaches are remarkable for early diagnosis and treatment, in these contexts, the hybridization among feature selection approaches and ML classification algorithms resulting several ML models. However, no intersection process can be leveraged to diagnose ASD patients based on hybridization models. In addition, the selection process for the best hybrid model based on multievaluation metrics simultaneously is a challenging task that falls under the multicriteria decision-making theory. Therefore, this study suggested using a new framework that can handle methodology in the context of used multimedical test features and sociodemographic characteristic features.

## Figures and Tables

**Table 1:** Description dataset used and information extraction from systematic literature review.

Ref.	Dataset resource/availability	Number of the dataset used	Dataset size	Number of attributes/features	Dataset area	Associated task
[16]	UCI repository	3 types	1-704,2-292,3-104	21	Questionnaires	Classification
[49]	ASDTests application	3 types (1-children, 2-adult, 3-adolescents)	1452	20	Questionnaires	Classification
[59]	NDAR repository	1	1534	13	Sociodemographic & medical	Classification
[48]	UCI repository	1	704	10	N/A	Classification
[19]	NDAR repository	12 (chose 4 only)	N/A	150 use (21)	Questionnaires	Classification
[51]	https://www.kaggle.com/fabdelja/autism-screening-for-toddlers	1	1054	17	Questionnaires	Classification
[41]	N/A	1	2400 (use 1034)	5 cluster	N/A	Clustering
[62]	UCI repository	1	702	21	Questionnaires	Classification
[4]	Kaggle and UCI repository	1	1054	18	Questionnaires	Classification
[1]	Autism Barta application	1	642	23 (use8)	Questionnaires	Classification
[53]	UCI repository	3 types (1-children, 2-adult, 3-adolescents)	1-292,2-704,3-104	21 (use 16)	Questionnaires	Classification
[61]	UCI repository	1	292	19	Questionnaires	Classification
[63]	https://archive.ics.uci.edu/ml/datasets/Autism+Screening+Adult#	1	704 (use 699)	21 (use 19)	Questionnaires	Classification
[47]	Autism clinic of the Shanghai Mental Health Center	1	122	27	Questionnaires	Classification
[46]	N/A	1	N/A	21	Questionnaires	Classification
[57]	ASDTests application	3 types (1-children, 2-adult, 3-adolescents)	1-292,2-704,3-104	20	Questionnaires	Classification
[64]	1-UCI repository 2- from a webpage publicized by a professor of data analytics	2 types	1-704 (use 609) 2-1118 (use 1117)	1-21 2-24	Questionnaires	Classification
[15]	Kaggle and UCI repository	4 types (1-toddlers, 2-child, 3-adult, 4-adolescent)	1-1054 2-248 3-609 4-98	20	Questionnaires	Classification
[64]	UCI repository	1	704	21 (use 17)	Questionnaires	Classification
[54]	UCI repository	1	702	19	Questionnaires	Classification
[14]	Kaggle and UCI repository	4 types (1-toddlers, 2-child, 3-adult, 4-adolescent)	2009	21 (use19)	Questionnaires	Classification
[60]	Kaggle	1	N/A	20	Questionnaires	Classification
[55]	From another paper “Autism Spectrum Disorder Screening: Machine Learning Adaptation and DSM-5 Fulfillment”	1	704	19	Questionnaires	Classification
[44]	UCI repository	3 types (1-children, 2-adult, 3-adolescents)	1-292,2-704,3-104	21	Questionnaires	Classification
[5]	UCI repository	3 types (1-children, 2-adult, 3-adolescents)	N/A	19 (use 12)	Questionnaires	Classification
[42]	From another paper “Autism Spectrum Disorder Screening: Machine Learning Adaptation and DSM-5 Fulfillment	1	704	20	Questionnaires	Classification
[45]	Collected from different sources	1	2000	40	Questionnaires	Classification
[68]	ASDTests application	1	1054	18	Questionnaires	Classification
[50]	Kaggle and UCI repository	3 types (1-children, 2-adult, 3-adolescents)	1-292,2-704,3-104	21	Questionnaires	Classification
[56]	UCI repository	1	292	21	Questionnaires	Classification
[67]	ASDTests application	1	1-292,2-704,3-104	23 use (16)	Questionnaires	Classification
[13]	Kaggle and UCI repository	Part 1: -3 types 1-children 2-adult 3-adolescents	1-292,2-704,3-104	21	Questionnaires	Classification
	Collected from an institute of special education for the people with special needs, and 150 data of non-ASD cases were collected through field visit to different schools and shopping malls	Part 2: -use real data	250			
[52]	1. http://archive.ics.uci.edu/ml//datasets/Autistic+Spectrum+Disorder+Screening+Data+for+Children++2. https://archive.ics.uci.edu/ml/datasets/Autistic+Spectrum+Disorder+Screening+Data+for+Adolescent+++3. https://archive.ics.uci.edu/ml/datasets/Autism+Screening+Adult	3 types (1-children, 2-adult, 3-adolescents)	1-292 use (248),2-704 use (609),3-104 use (98)	20	Questionnaires	Classification
[43]	UCI repository	1	290 use (241)	21(use 4 question)	Questionnaires	Classification
[58]	1. http://archive.ics.uci.edu/ml//datasets/Autistic+Spectrum+Disorder+Screening+Data+for+Children++ . 2. https://archive.ics.uci.edu/ml/datasets/Autistic+Spectrum+Disorder+Screening+Data+for+Adolescent+++ . 3. https://archive.ics.uci.edu/ml/datasets/Autism+Screening+Adult	3 types (1-children, 2-adult, 3-adolescents)	1-292,2-704,3-104	21	Questionnaires	Classification
[18]	Kaggle repository	1	1054	23	Questionnaires	Classification
[65]	UCI repository	3 types (1-children, 2-adult, 3-adolescents)	1-292,2-704,3-104	21	Questionnaires	Classification
[6]	Autism therapy counseling and help (CATCH, Bhubaneswar, India)	N/A	500	35	N/A	Classification
[39]	Data not publicly available due to medical confidentiality but are available from the first author on reasonable request pending the approval of the coauthors	1-adult, 2-adolescents	673	31 (use 5,11,12,31)	Questionnaires	Classification
[40]	Association of parents and friends for the support and defense of the rights of people with autism	1	N/A	ADIR = 19 ADOS = 11	Questionnaires	Classification

**Table 2:** ML methods and evaluation metrics results extracted from the literature.

Ref.	Methods used	Evaluation performance metrics							
		Accuracy	Specificity	Sensitivity/recall	F1	AUC	Precision	TPR	FPR
[16]	SVM	98.11	0.9574	0.8888					
	NB	96.22	0.93610	0.9696					
	CNN	99.53	1.0	0.9757					
	LR	96.69	0.9575	0.9696					
	KNN	95.75	0.9148	0.9696					
	ANN	97.64	0.9787	0.9757					
[59]	Decision tree	91.1	0.71	0.91					
[48]	SVM	100%							
	NB	97.017%							
	Decision table	100%							
[19]	Decision tree	FM = 89.1, SM = 89.3						FM = 89.4, SM = 90.4	FM = 70.8, SM = 66.6
	AD Tree	FM = 88.7, SM = 88.4						FM = 89.0, SM = 89.2	FM = 81.8, SM = 72.3
	CDT	FM = 88.2, SM = 90.5						FM = 89.3, SM = 89.6	FM = 67.5, SM = 73.3
	J48	FM = 88.8, SM = 91.7						FM = 89.7, SM = 94.8	FM = 73.0, SM = 71.7
	LAD Tree	FM = 89.2, SM = 87.55						FM = 89.8, SM = 89.6	FM = 63.3, SM = 55.0
[62]	DENN		0.99	0.99	0.99				
	NN		0.94	0.94	0.94				
	RF		0.92	0.91	0.91				
	SVM		0.73	0.73	0.73				
	Gradient boosting		0.85	0.85	0.85				
[4]	SVM	0.83		0.88	0.88	0.88	0.89		
	NB	0.89		0.84	0.91	0.91	1.0		
	RF	0.93		1.0	0.96	0.96	0.92		
	KNN	0.98		0.97	0.99	0.99	1.0		
[1]	J48	98.44		0.984	0.984		0.984		
	LMT	98.44		0.984	0.984		0.984		
	DS	97.82		0.978	0.977		0.979		
	REP Tree	97.66		0.977	0.976		0.977		
	NP Tree	97.98		0.980	0.979		0.980		
[53]	DT	Child = 82.76, adolescent = 83.87, adult = 91		Overall % AVERAG = 84.71			Overall % AVERAG = 86.45		
	NB	Child = 93.1, adolescent = 80.65, adult = 97.16		Overall % AVERAG = 88.98			Overall % AVERAG = 89.25		
	KNN	Child = 86.21, adolescent = 87.1, adult = 93.36		Overall % AVERAG = 88.90			Overall % AVERAG = 91.87		
	RT	Child = 67.82, adolescent = 77.4, adult = 72.99		Overall % AVERAG = 62.72			Overall % AVERAG = 56.43		
	Deep learning	Child = 96.55, adolescent = 93.55, adult = 99.05		Overall % AVERAG = 96.49			Overall % AVERAG = 96.10		
[61]	LDA	0.9080	0.8667	0.9524	0.9091		0.8696		
	KNN	0.8851	0.8000	0.9762	0.8913		0.8200		
[63]	RF	0.9571	0.9821	0.8571					
[47]	DNN	86.96			Nonautism = 0.8000, mildautism = 0.8235, severe autism = 0.9474				
	OVR-SVM	56.52			Nonautism = 0.571, mildautism = 0.6154, severe autism = 0.4615				
	CART	60.87			Nonautism = 0.545, mildautism = 0.5556, severe autism = 0.7059				
[46]	LR			0.97	0.97		0.97		
[57]	DT	Child = 0.8226, adolescent = 0.80, adult = 0.8798	Child = 0.8710, adolescent = 0.4444, adult = 0.9302	Child = 0.7742, adolescent = 0.9375, adult = 0.7593	Child = 0.8136, adolescent = 0.8571, adult = 0.7885	Child = 0.8225, adolescent = 0.9375, adult = 0.8447			
	RF	Child = 0.8226, adolescent = 0.88, adult = 0.9180	Child = 0.8387, adolescent = 0.8889, adult = 0.9690	Child = 0.8065, adolescent = 0.8750, adult = 0.8148	Child = 0.8197, adolescent = 0.900, adult = 0.8544	Child = 0.9433, adolescent = 0.9444, adult = 0.9812			
	RF (hyperparameter)	Child = 0.9032, adolescent = N/A, adult = 0.9727	Child = 0.8701, adolescent = N/M, adult = 0.9922	Child = 0.9355, adolescent = N/A, adult = 0.9259	Child = 0.9062, adolescent = N/A, adult = 0.9524	Child = 0.9977, adolescent = N/A, adult = 0.9977			
	LR	Child = 0.9032, adolescent = 0.92, adult = 0.9836	Child = 0.8710, adolescent = 0.7778, adult = 0.9845	Child = 0.9354, adolescent = 1.00, adult = 0.9814	Child = 0.9062, adolescent = 0.9412, adult = 0.9725	Child = 0.9865, adolescent = 0.9931, adult = 0.9959			
	SVM	Child = 0.9677, adolescent = 0.80, adult = 0.9235	Child = 0.9355, adolescent = 0.4444, adult = 0.9690	Child = 1.00, adolescent = 1.00, adult = 0.8148	Child = 0.9688, adolescent = 0.8649, adult = 0.8627	Child = 0.9896, adolescent = 0.9861, adult = 0.9886			
	ANN	Child = 0.9516, adolescent = 0.76, adult = 0.9891	Child = 0.9355, adolescent = 0.8125, adult = 0.9922	Child = 0.9677, adolescent = 0.9815, adult = 0.9815	Child = 0.9508, adolescent = 0.8125, adult = 0.9815	Child = 0.9896, adolescent = 0.9861, adult = 0.9887			
[64]	DNN	Dataset1 = 90.4, Dataset2 = 96.08	Dataset1 = 100, Dataset2 = 97.32	Dataset1 = 97.89, Dataset2 = 92.68					
	SVM	Dataset1 = 95.24, Dataset2 = 95.08	N/A	N/A					
[15]	ANN	Toddlers = 0.9896, child = 0.9589, adolescent = 0.903, adult = 0.9901	Toddlers = 0.9886, child = 0.9593, adolescent = 0.8948, adult = 0.9964	Toddlers = 0.9886, child = 0.9589, adolescent = 0.9038, adult = 0.9901	Toddlers = 0.9896, child = 0.9589, adolescent = 0.9038, adult = 0.9901	Toddlers = 0.9891, child = 0.9591, adolescent = 0.8993, adult = 0.9932			
	RNN	Toddlers = 0.9943, child = 0.9726, adolescent = 0.884, adult = 0.9673	Toddlers = 0.9924, child = 0.9721, adolescent = 0.8823, adult = 0.9110	Toddlers = 0.9943, child = 0.9726, adolescent = 0.8846, adult = 0.9673	Toddlers = 0.9943, child = 0.9726, adolescent = 0.8851, adult = 0.9666	Toddlers = 0.9933, child = 0.9723, adolescent = 0.8835, adult = 0.9392			
	DT	Toddlers = 0.9175, child = 0.8938, adolescent = 0.759, adult = 0.9062	Toddlers = 0.8885, child = 0.8938, adolescent = 0.6817, adult = 0.8450	Toddlers = 0.9175, child = 0.8938, adolescent = 0.7596, adult = 0.9062	Toddlers = 0.9174, child = 0.8938, adolescent = 0.7482, adult = 0.9058	Toddlers = 0.9030, child = 0.8938, adolescent = 0.7207, adult = 0.8756			
	ELM	Toddlers = 0.9231, child = 0.8973, adolescent = 0.826, adult = 0.9190	Toddlers = 0.8860, child = 0.8965, adolescent = 0.8363, adult = 8531	Toddlers = 0.9231, child = 0.8973, adolescent = 0.8269, adult = 0.9190	Toddlers = 0.9227, child = 0.8972, adolescent = 0.8285, adult = 0.9181	Toddlers = 0.9046, child = 0.8969, adolescent = 0.8316, adult = 0.8860			
	GB	Toddlers = 0.9782, child = 0.9315, adolescent = 0.8750, adult = 0.9659	Toddlers = 0.9665, child = 0.9318, adolescent = 0.8335, adult = 0.9306	Toddlers = 0.9782, child = 0.9315, adolescent = 0.8750, adult = 0.9659	Toddlers = 0.9781, child = 0.8915, adolescent = 0.8725, adult = 0.9656	Toddlers = 0.9723, child = 0.9317, adolescent = 0.8542, adult = 0.9482			
	KNN	Toddlers = 0.9488, child = 0.8904, adolescent = 0.8077, adult = 0.9432	Toddlers = 0.9398, child = 0.8953, adolescent = 0.7130, adult = 0.9289	Toddlers = 0.9488, child = 0.8904, adolescent = 0.8077, adult = 0.9432	Toddlers = 0.9490, child = 0.8901, adolescent = 0.7927, adult = 0.9436	Toddlers = 0.9443, child = 0.8929, adolescent = 0.7604, adult = 0.9360			
	LR	Toddlers = 1.0, child = 0.9932, adolescent = 0.951, adult = 0.9986	Toddlers = 1.0, child = 0.9927, adolescent = 0.9346, adult = 0.9961	Toddlers = 1.0, child = 0.9932, adolescent = 0.9519, adult = 0.9986	Toddlers = 1.0, child = 0.9931, adolescent = 0.9516, adult = 0.9986	Toddlers = 1.0, child = 0.9929, adolescent = 0.9433, adult = 0.9974			
	MLP	Toddlers = 0.9991, child = 0.9863, adolescent = 0.942, adult = 0.9957	Toddlers = 0.9996, child = 0.9858, adolescent = 0.9199, adult = 0.9951	Toddlers = 0.9991, child = 0.9863, adolescent = 0.9423, adult = 0.9957	Toddlers = 0.9991, child = 0.9863, adolescent = 0.9417, adult = 0.9957	Toddlers = 0.9993, child = 0.9861, adolescent = 0.9311, adult = 0.9954			
	NB	Toddlers = 0.9431, child = 0.8664, adolescent = 0.855, adult = 0.9418	Toddlers = 0.9152, child = 0.8635, adolescent = 0.8380, adult = 0.9150	Toddlers = 0.9431, child = 0.8664, adolescent = 0.8558, adult = 0.9418	Toddlers = 0.9428, child = 0.8661, adolescent = 0.8554, adult = 0.9419	Toddlers = 0.9291, child = 8650, adolescent = 0.8469, adult = 0.9284			
	RF	Toddlers = 0.9592, child = 0.9110, adolescent = 0.894, adult = 0.9588	Toddlers = 0.9343, child = 0.9098, adolescent = 0.8630, adult = 0.9112	Toddlers = 0.9592, child = 0.9110, adolescent = 0.8942, adult = 0.9588	Toddlers = 0.9590, child = 0.9109, adolescent = 0.8928, adult = 0.9583	Toddlers = 0.9468, child = 0.9104, adolescent = 0.8786, adult = 0.9350			
	SVM	Toddlers = 0.9753, child = 0.9452, adolescent = 0.894, adult = 0.9716	Toddlers = 0.9568, child = 0.9446, adolescent = 0.8545, adult = 0.9393	Toddlers = 0.9753, child = 0.9452, adolescent = 0.8942, adult = 0.9716	Toddlers = 0.9752, child = 0.9452, adolescent = 0.8921, adult = 0.9713	Toddlers = 0.9661, child = 0.9449, adolescent = 0.8744, adult = 0.9555			
	XGB	Toddlers = 0.9820, child = 0.9555, adolescent = 0.913, adult = 0.9659	Toddlers = 0.9767, child = 0.9556, adolescent = 0.8755, adult = 0.9406	Toddlers = 0.9820, child = 0.9555, adolescent = 0.9135, adult = 0.9659	Toddlers = 0.9820, child = 0.9555, adolescent = 0.9117, adult = 0.9658	Toddlers = 0.9793, child = 0.9555, adolescent = 0.8945, adult = 0.9533			
[54]	KNN	67.5564							
	LR	72.0238							
	SVM	70.5952							
	LDA	72.2024							
	NB	70.7769							
	Classification and regression tree	69.1667							
[60]	WOEM	99	98	98					
	SLFN(ELM)	96	96	96					
	SVM	94	96.5	96.5					
	ANN	90	95	95					
	KNN	95	97	97					
[55]	DT	2K‐fold = 100, 3K‐fold = 100, 4K‐fold = 100, 5K‐fold = 100, 6K‐fold = 100, 7K‐fold = 100, 8K‐fold = 100‐‐fold = 100							
	LDA	2K‐fold = 96.3, 3K‐fold = 96.7, 4K‐fold = 96.7, 5K‐fold = 96.7, 6K‐fold = 96.6, 7K‐fold = 96.9, 8K‐fold = 96.7, 9K‐fold = 96.9, 10K‐fold = 96.9							
	LR	2K‐fold = 99.7, 3K‐fold = 99.7, 4K‐fold = 99.6, 5K‐fold = 99.6, 6K‐fold = 99.6, 7K‐fold = 99.6, 8K‐fold = 99.6, 9K‐fold = 99.6, 10K‐fold = 99.6							
	SVM	2K‐fold = 99.3, 3K‐fold = 99.3, 4K‐fold = 98.9, 5K‐fold = 99.3, 6K‐fold = 99.4, 7K‐fold = 99.6, 8K‐fold = 99.4, 9K‐fold = 99.9, 10K‐fold = 99.9							
	KNN	2K‐old = 92.8, 3K‐fold = 94, 4K‐fold = 93, 5K‐fold = 93.8, 6K‐fold = 92.9, 7K‐fold = 933, 8K‐fold = 94.2, 9K‐fold = 93.9, 10K‐fold = 92.9							
[44]	RF	Child = 1.0, adolescent = 0.976, adult = 0.992		Child = 0.993, adolescent = 0.976, adult = 0.993			Child = 1.0, adolescent = 0.977, adult = 0.993		
	LR	Child = 0.923, adolescent = 0.881, adult = 0.94		Child = 0.923, adolescent = 0.881, adult = 0.94			Child = 0.923, adolescent = 0.908, adult = 0.94		
	NB	Child = 0.983, adolescent = 1.0, adult = 0.986		Child = 0.983, adolescent = 1.0, adult = 0.986			Child = 0.984, adolescent = 1.0, adult = 0.986		
	MCR with average of probabilities	Child = 0.932, adolescent = 0.93, adult = 0.996		Child = 0.932, adolescent = 0.929, adult = 0.996			Child = 0.932, adolescent = 0.939, adult = 0.996		
	MCR with majority voting	Child = 0.983, adolescent = 1.0, adult = 1.0		Child = 0.983, adolescent = 1.0, adult = 1.0			Child = 0.984, adolescent = 1.0, adult = 1.0		
[5]	J48	Child = 92, adolescent = 81adult = 93, complete = 92	Child = 93, adolescent = 83, adult = 86, complete = 87	Child = 91, adolescent = 76, adult = 96, complete = 94					
	RF	Child = 93, adolescent = 92, adult = 96, complete = 95	Child = 92, adolescent = 91, adult = 93, complete = 94	Child = 95, adolescent = 94, adult = 93, complete = 92					
	Bayes	Child = 96, adolescent = 92, adult = 97, complete = 96	Child = 95, adolescent = 91, adult = 93, complete = 92	Child = 97, adolescent = 95, adult = 98, complete = 98					
	Adaboost	Child = 90, adolescent = 89, adult = 94, complete = 92	Child = 86, adolescent = 86, adult = 87, complete = 84	Child = 95, adolescent = 95, adult = 93, complete = 92					
	PART	Child = 92, adolescent = 85, adult = 94, complete = 95	Child = 90, adolescent = 86, adult = 88, complete = 94	Child = 90, adolescent = 86, adult = 87.5, complete = 94					
	ANN	Child = 92, adolescent = 87, adult = 96, complete = 95.5	Child = 90, adolescent = 84, adult = 94, complete = 95	Child = 90, adolescent = 84, adult = 94.5, complete = 95					
	SVM	Child = 94, adolescent = 93, adult = 97, complete = 97	Child = 94, adolescent = 90, adult = 95, complete = 96	Child = 94, adolescent = 90, adult = 95, complete = 96					
	AttSelclasss	Child = 93, adolescent = 85, adult = 96, complete = 97	Child = 97.5, adolescent = 86, adult = 99, complete = 98	Child = 97, adolescent = 86, adult = 99, complete = 98					
[42]	FARF (combined Firefly-Random Forest)	94.32			35.10				
	RF	90.78			34.09				
[45]	ANN	83	83.5	84	85		83		
	SVM	91	86.5	86	86		86		
	IANFIS	98	90	91	92		89		
[56]	SVM-NP	95.547		0.940	0.956		0.973		
	SVM-PK	100		1.00	1.00		1.00		
	SVM-PUK	100		1.00	1.00		1.00		
	SVM-RBF	99.315		0.993	0.993		0.993		
[58]	SVM	*A*dolescent = 59.6154, child = 53.0822, adult = 69.7443							
	Active pruning rules (APR)	Adolescent = 66.3462, child = 60.9589, adult = 73.7216							
	RKFNN	Adolescent = 72.1154, child = 71.2329, adult = 80.2257							
[65]	BFNNRELU	Child = 98.73, adolescent = 94.32, adult = 97.28	Child = 0.9678, adolescent = 0.9380, adult = 0.9601	Child = 0.9716, adolescent = 0.9571, adult = 0.9601		Child = 0.9697, adolescent = 0.9475, adult = 0.9559			
	ANFAND	Child = 73.63, adolescent = 81.50, adult = 95.57	Child = 0.7333, adolescent = 0.9259, adult = 0.8976	Child = 0.5236, adolescent = 0.6758, adult = 0.8976		Child = 0.6577, adolescent = 0.8061, adult = 0.9379			
	SVM	Child = 98.67, adolescent = 89.26, adult = 94.21	Child = 0.9872, adolescent = 0.8745, adult = 0.8946	Child = 0.9954, adolescent = 0.9452, adult = 0.8946		Child = 0.9913, adolescent = 0.9098, adult = 0.9350			
	MLP	Child = 99.05, adolescent = 90.28, adult = 99.91	Child = 0.9952, adolescent = 0.9402, adult = 0.9977	Child = 0.9860, adolescent = 0.8451, adult = 0.9977		Child = 0.9906, adolescent = 0.8926, adult = 0.9986			
	NB	Child = 99.82, adolescent = 93.35, adult = 96.51	Child = 1.0, adolescent = 0.9947, adult = 0.9846	Child = 1.0, adolescent = 0.9152, adult = 0.9846		Child = 1.0, adolescent = 0.9459, adult = 0.9781			
	C4.5	Child = 94.86, adolescent = 89.13, adult = 91.86	Child = 0.7854, adolescent = 0.8851, adult = 0.7500	Child = 0.9987, adolescent = 0.8926, adult = 0.7500		Child = 0.8920, adolescent = 0.8885, adult = 0.8750			
	RNT	Child = 91.61, adolescent = 87.88, adult = 95.30	Child = 0.9108, adolescent = 0.9313, adult = 0.9065	Child = 0.9210, adolescent = 0.7963, adult = 0.9065		Child = 0.9259, adolescent = 0.8638, adult = 0.9382			
[40]	MCFM	0.842			0.843				
	SVM	0.833			0.833				
	RF	0.851			0.852				
	NB	0.865			0.865				

FM: family medical history; SM: subject medical history.
